# T146. AROUSAL AFFECTS DIFFERENTIALLY FIRST-EPISODE PSYCHOSIS PATIENTS AND CONTROL SUBJECTS’ DEFAULT MODE NETWORK FUNCTIONING DURING MOVIE VIEWING

**DOI:** 10.1093/schbul/sby016.422

**Published:** 2018-04-01

**Authors:** Teemu Mantyla, Jussi Alho, Eva Rikandi, Tuula Kieseppä, Jaana Suvisaari, Tuukka Raij

**Affiliations:** 1 National Institute for Health and Welfare; 2 Aalto University School of Science; 3 National Institute for Health and Welfare, University of Helsinki; 4 University of Helsinki, Helsinki University Hospital; 5 Aalto University School of Science, University of Helsinki, Helsinki University Hospital

## Abstract

**Background:**

Functional alterations of the default mode network (DMN) are frequently reported in psychotic disorders, but the functional role of these alterations remains poorly known. In addition to previous studies that have applied different types of tasks or recorded resting-state neuroimaging data, there has recently been more interest in the use of movie stimuli in studying brain functioning in patient populations, because this could provide a more naturalistic account of brain functioning in real life-like situations.

**Methods:**

Seventy-one first-episode psychosis (FEP) patients (mean age = 26.0 yrs, 47 (66%) males) and 57 controls (mean age = 26.86 yrs, 24 (42%) males) from the Helsinki Early Psychosis Study watched scenes from the movie Alice in Wonderland (Tim Burton, 2010) during 3 T fMRI-BOLD imaging. We used intersubject correlation (ISC) analysis, in which the correlation between voxel-wise BOLD time series in every within-group pair of subjects is calculated. In this study, time-windowed ISC was calculated with a 10-TR (time of repetition, 1.8 s) window with 1-TR steps over the fMRI time series. In each ISC window, a two-sample t test was performed to obtain a t-statistic time series of differences between the groups. An independent group of control subjects (n = 17, 10 males, mean age 26.5 yrs) rated how emotionally arousing the currently seen events of the stimulus are, producing a time-varying rating used as a regressor. General linear model was used to identify brain regions where the t-statistic time series covaries with the arousal rating. To make the interpretation of results less ambiguous, the arousal rating was divided into high and low arousal regressor by z scoring the rating and taking only the positive and negative values, respectively. Nonparametric clusterwise permutation test was used for statistical inference (cluster-defining threshold of p = 0.05, familywise error corrected threshold of p = 0.05, number of permutations = 5000). Furthermore, by using an experience-sampling setup during the same brain-scanning session, a partially overlapping sample of participants reported how emotionally aroused they were feeling during scanning.

**Results:**

The results show significant correlation between the t-statistic time series and low arousal regressor, especially in the DMN including the anterior and posterior cingulate cortex, medial prefrontal cortex, precuneus, and bilateral lateral temporoparietal regions. Closer inspection reveals that during moments of low arousal in the movie stimulus, the ISC of healthy controls goes up but the ISC of patients does not. In the experience-sampling portion of the study, the patients reported more arousal than the control subjects.

**Discussion:**

Intersubject correlation in the DMN depended differentially on arousal in FEP patients and control subjects. More specifically, during moments when the stimulus was rated less emotionally arousing, control subjects’ DMN functioning synchronized more while the patients’ did not. In connection with the difference in reported arousal during the same imaging session, our findings provide preliminary evidence for a contribution of arousal on the functional alterations of the DMN and suggest that this may be related to higher baseline arousal in the patients. Higher arousal and the related distortion of high order integrative functioning that characterizes DMN could contribute to the pathogenesis of psychosis.

